# The Cost-Effectiveness of Requesting a Complete Blood Count (CBC) in the Management of COVID-19 in Saudi Arabia

**DOI:** 10.3390/healthcare10091780

**Published:** 2022-09-15

**Authors:** Anwar A. Sayed

**Affiliations:** 1 Department of Medical Microbiology and Immunology, Taibah University, Medina 42353, Saudi Arabia; anwar.sayed13@imperial.ac.uk; 2 Department of Surgery and Cancer, Imperial College London, London SW7 2AZ, UK

**Keywords:** CBC, cost-effectiveness, COVID-19, management, Saudi Arabia

## Abstract

Background: Since the beginning of the COVID-19 pandemic, studies have attempted to determine the value of the different laboratory investigations. This study aims to assess the cost-effectiveness of requesting a complete blood count (CBC) for COVID-19 patients, as opposed to ordering a COVID-19 antibody titer in Saudi Arabia. Methods: The prices of a CBC, COVID-19 PCR, and antibody titer were obtained from 40 healthcare establishments in Saudi Arabia, between January and February 2022. Results: Requesting a CBC is significantly cheaper than requesting a COVID-19 antibody titer, which was available in almost all of the establishments, as compared to COVID-19 PCR and antibody titer testing. The investigation prices did not differ significantly between hospitals and private laboratories, nor across cities in Saudi Arabia. Conclusions: CBC, which provides valuable information on the patient’s condition and prognosis, is a cost-effective and widely available tool for managing COVID-19. The price and availability of CBC warrant it to be included in the COVID-19 management protocols.

## 1. Introduction

COVID-19 has taken the world by storm, causing many countries to take extensive measures to limit its impact [1]. Since the beginning of the latest global pandemic, COVID-19, healthcare providers and scientists have raced to determine the ideal way of managing COVID-19 cases. The management of COVID-19 cases expands from detection, forming a diagnosis, and establishing baseline parameters, to determining the current health status of a patient, all the way to prevention and treatment via pharmacotherapy.

A complete blood count (CBC), also known as a hemogram [2], is a common laboratory investigation that is used in a wide range of conditions. CBC shows basic hematological indices of red blood cells (RBC), e.g., RBC count, hemoglobin levels, and mean corpuscular volume; white blood cells (WBC), e.g., WBC count and differential count (granulocyte and lymphocyte count); and platelets. CBC has become a routine laboratory investigation due to its value in clinical practice and is requested at the beginning of the management of almost any conditions. However, its value in COVID-19 has not been fully appreciated.

Using a CBC, a physician could estimate the neutrophil-to-lymphocyte ratio (NLR), which is calculated by dividing the absolute neutrophil count by the lymphocyte count. Based on a normal neutrophil range between 4.28 and 4.64 × 10^3^/μL, and a lymphocyte normal range between 1.95 and 2.1 × 10^3^/μL, a normal NLR would range between 2.18 and 2.21 [3]. NLR has been the focus of many studies on COVID-19, and was found to be a reliable predictor of COVID-19 severity [3,4], hospitalization [5], and mortality [6,7,8]. Other tested parameters have been indicated in the pathogenesis of COVID-19, such as the positivity of a COVID-19 Polymerase Chain Reaction (PCR) test after a certain number of days, or COVID-19 antibody titers, which reflect immunity against the virus [9].

Although a CBC is important to calculate the NLR, the cost-effectiveness of requesting a CBC, as opposed to COVID-19 PCRs and antibody titers, for COVID-19 patients has not been previously analyzed in Saudi Arabia (SA). Assessing the cost-effectiveness of requesting a CBC in the context of COVID-19 would better inform healthcare policymakers about including a CBC in the management of COVID-19 conditions.

## 2. Materials and Methods

This is a cross-sectional study that was conducted between January and February 2022. The prices of CBC, COVID-19 PCR, and anti-COVID-19 antibody titers at the included healthcare facilities were retrieved either from their commercial webpage or by directly contacting the establishment. Prices were obtained in Saudi Arabia Riyals (SAR) and were converted to US dollars (USD) by using the fixed exchange rate of 1 USD = 3.75 SAR. The prices of laboratory investigations were collected from private hospitals and laboratories in SA. Exclusion criteria were unavailability of data or non-response when contacted.

Descriptive statistics were used to report the prices of the different tests based on their distribution. A Shapiro–Wilk test was used to determine the data normality, which was shown to be of non-parametric distribution. The median and interquartile ranges (IQR) were used to report the prices. Mann–Whitney U and Kruskal–Wallis tests were used to compare 2 independent groups or more, respectively. A *p*-value of less than 0.05 was considered statistically significant. Data analysis was carried out using GraphPad Prism version 9.1 (GraphPad Software, San Diego, CA, USA).

The study did not include human or animal subjects, and therefore, no IRB ethical approval was required for this study.

## 3. Results

Fifty healthcare facilities were initially included in this study; however, 11 were excluded, as described in the Materials and Methods. The majority of the included facilities were private laboratories (n = 34). These facilities were located in different cities across SA, including Madinah, Jeddah, Riyadh, and the Eastern Province, as shown in Table 1.

**Figure 1:**
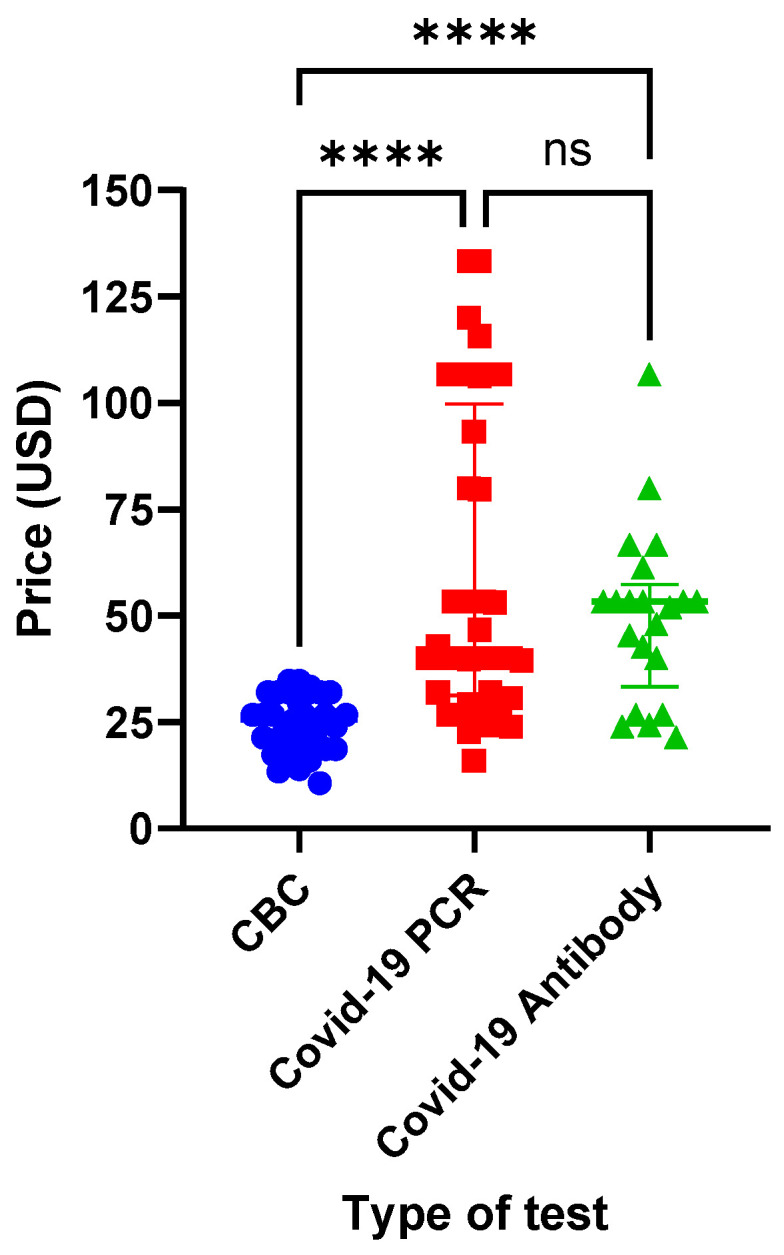
A comparison between the prices of the different investigations. The figure demonstrates a CBC (blue circles) to have a significantly lower price as compared to COVID-19 PCR tests (red squares) and antibody titers (green triangles). Prices are presented in USD. **** denotes a *p*-value of <0.0001. CBC: complete blood count; ns: non-significant; USD: United States dollars.

CBCs were the cheapest laboratory investigation, with a median price of USD 25.60 (IQR: 18.67–32), followed by a COVID-19 PCR test, with a median price of USD 40 (IQR: 31.33–99.87), and lastly, COVID-19 antibody titers, with a median price of 53.33 (IQR: 33.33–57.33), as shown in Figure 1. CBC prices were significantly lower than those of both COVID-19 PCR tests and antibody titers (*p*-value < 0.0001).

**Figure 2:**
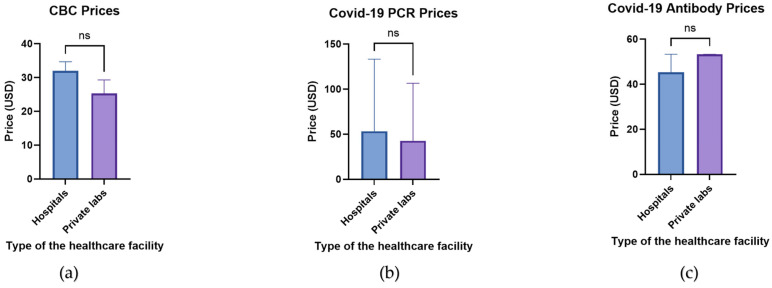
A comparison between the investigation prices at hospitals and private laboratories. The bar charts represent the prices of (**a**) CBC at hospitals (blue bars) as compared to private laboratories (purple labs). (**b**) The prices of COVID-19 PCR tests and (**c**) anti-COVID-19 antibody titers are also demonstrated. The data demonstrate median values and IQR. ns: non-significant; USD: United States dollars.

In order to determine whether the type of healthcare establishment affects the prices, a comparison was made between the prices of each investigation between hospitals and private laboratories (Figure 2). The prices of both the CBC (Figure 2a) and COVID-19 PCR testing (Figure 2b) were more expensive at hospitals, as compared to private laboratories, whereas the prices of COVID-19 antibody titers (Figure 2c) were cheaper at hospitals. However, these differences were not statistically significant, which demonstrates that the type of medical establishment did not affect the studied laboratory investigations.

**Figure 3:**
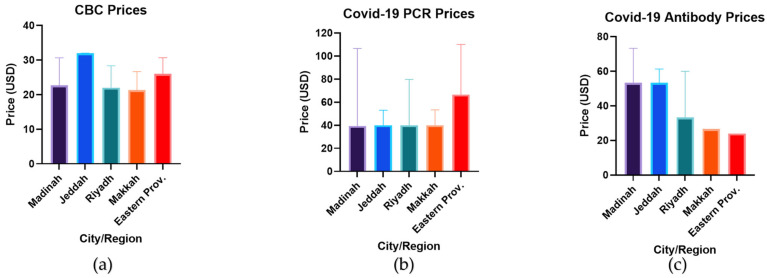
A comparison between the investigation prices based on the location of the medical establishment. The bar charts represent the prices of (**a**) CBC in Madinah (dark purple), Jeddah (blue), Riyadh (green), Makkah (orange), and the Eastern Province (red), and (**b**) the prices of COVID-19 PCR tests and (**c**) anti-COVID-19 antibody titers across the different cities. The data demonstrate median values and IQR. USD: United States dollars.

As the healthcare facilities included in the study are from different regions/cities of SA, the prices of each investigation were compared based on the location of these facilities. Makkah was found to have the lowest prices of both CBC and COVID-19 PCR tests (Figure 3a,b), whereas the Eastern Province had the cheapest anti-COVID-19 antibody titers (Figure 3c). Although the investigation prices varied between cities, these differences were not statistically significant (*p* value > 0.05), further showing the homogeneity of prices across SA.

## 4. Discussion

CBC is one of the basic investigations that many physicians routinely request upon meeting a new patient. This study aimed to assess its cost-effectiveness in the management of COVID-19 cases, more specifically in SA. The value of CBC in the context of COVID-19 lies in its provision of NLR. Although abnormally high NLR is of considerable sensitivity and specificity in diagnosing COVID-19 [3], COVID-19 PCR tests remain the gold standard in diagnosing COVID-19 cases [10]. After the diagnosis of COVID-19 cases, physicians are faced with a common dilemma, which is whether to admit patients to the hospital and, if so, to the wards or the intensive care unit (ICU). NLR, which is derived from CBCs, has been demonstrated in multiple studies to be an accurate predictor of severe COVID-19 [3,11,12,13], hospitalization [5], and mortality [6,7,8]. Comparatively, after the diagnosis of COVID-19, COVID-19 PCR tests and anti-COVID-19 antibody titers only reflect the disease (virus) presence and the level of immunity against it, respectively. The value of antibody titers in the context of COVID-19 is still debatable, with studies demonstrating conflicting results regarding its correlation with disease severity [14,15,16].

The low price of CBC is not the sole reason for its cost-effectiveness when managing COVID-19 cases. CBC is widely available, as almost all the healthcare facilities in this study provide this laboratory investigation, whereas COVID-19 PCR and antibody titers are not as readily available. The availability of an investigation is a vital consideration when evaluating its cost-effectiveness, especially in remote areas and low-income countries. Additionally, although CBC can be used to calculate NLR, the usability of CBC extends beyond COVID-19 to be used to diagnose and monitor other comorbidities, e.g., anemia or immune thrombocytopenia [17]. However, the other tests are strictly limited to COVID-19 and are not of any direct value in detecting or monitoring any other condition.

Cost-effectiveness studies are widely conducted in a variety of conditions to assess the use of a specific medication, e.g., the use of fruquintinib in colorectal carcinoma [18], or the value of an added intervention, e.g., the use of adjuvant therapy in early-stage colon cancer [19]. However, cost-effectiveness studies focused entirely on CBC are lacking. A database search of PubMed for studies on the cost-effectiveness of CBC yielded a single study [20], and Google Scholar yielded only two studies [21,22] and a Master’s thesis [23]. Such a lack of studies makes this study the first of its kind to assess the cost-effectiveness of CBC in the context of COVID-19.

Typical cost-effectiveness studies are conducted based on a single cost of an investigation or treatment and then modeled in different scenarios to determine whether it is cost-effective or not. Here, the study is based on the latest prices of the studied investigations, making it more reliable and reflecting the current healthcare costs in SA.

Currently, the latest Saudi Ministry of Health COVID-19 management protocol does not include CBC as a prognostic tool for disease severity [24]. It relies on less common and less available investigations such as serum interleukin (IL)-6 and ferritin levels. The findings of this study, in addition to the vast literature supporting the use of NLR derived from a CBC, warrant its inclusion in the COVID-19 management protocol.

This study is not without limitations. The prices included in this study were collected from private hospitals and laboratories in SA. These prices may not reflect the actual price of the studied investigations, as these establishments are profitable businesses. Additionally, all of the prices were collected from healthcare facilities in a single country, SA. SA is classified as a high-income country with an estimated gross domestic product (GDP) per capita of USD 23,585.9 [25]. Hence, the investigations’ prices presented in this study may not be representative globally.

Future cost-effectiveness studies on the use of CBC in the context of COVID-19 could be extended to include countries of variable financial status, and other investigations that might of clinical significance in the management of COVID-19.

## 5. Conclusions

CBC is a cost-effective laboratory investigation that provides valuable information on the patient’s condition and prognosis when managing COVID-19 cases. The price and availability of CBC warrant its inclusion in the COVID-19 management protocols, rather than leaving it to the discretion of the treating physician.

## Figures and Tables

**Table 1:** The healthcare facilities included in the study.

Characteristics	Values
Type of healthcare facility	Hospitals (n = 5)
	Laboratories (n = 34)
Location of the facility	Madinah (n = 9) Jeddah (n = 8) Riyadh (n = 8) Makkah (n = 6) Eastern Province (n = 8)

## Data Availability

Not applicable.
